# Prevalence trends, population characteristics and treatment outcomes of tuberculosis combined with diabetes in Southwest China: a register-based retrospective study

**DOI:** 10.3389/fpubh.2024.1445857

**Published:** 2024-11-20

**Authors:** Deliang Kong, Yongping Xia, Xiangliu Wang, Yingtong Zhang, Jiyuan Zhong, Ting Zhang, Chengguo Wu, Jun Fan, Chuan Pu

**Affiliations:** ^1^School of Public Health, Chongqing Medical University, Chongqing, China; ^2^Institute of Tuberculosis Prevention and Treatment of Chongqing, Chongqing, China

**Keywords:** chronic disease epidemics, pulmonary tuberculosis, diabetes mellitus, comorbidity, adverse treatment outcomes

## Abstract

**Background:**

The global situation regarding the prevention and control of pulmonary tuberculosis (PTB) remains challenging. With the ongoing aging population and the increasing prevalence of chronic diseases, the epidemic of comorbid pulmonary tuberculosis and diabetes mellitus (PTB-DM) presents challenges to PTB control. We conducted this study given that current research on PTB-DM has primarily focused on clinical medicine and immunology, with limited reports on the true prevalence of population-wide PTB-DM in specific regions, as well as the heightened risk of PTB-DM co-prevalence due to aging and the high prevalence of DM in Chongqing, Southwest China.

**Methods:**

This retrospective study used PTB case data from the PTB Information Management System within the China Information System for Disease Control and Prevention (CISDP). The medical records of 112,592 PTB patients registered in Chongqing from 2016 to 2022 were extracted. After excluding patients with incomplete records, those not residing in Chongqing, and individuals still undergoing treatment, a total of 108,003 PTB patients were included in the study. The trend in PTB-DM incidence was analyzed using the Joinpoint regression model, and population and clinical characteristics of patients were described using frequencies (n) and percentages. Chi-squared test and Fisher’s exact tests was used to compare groups, and multivariable logistic regression model with stepwise backward elimination based on the Wald test was used to examine risk factors for adverse treatment outcomes.

**Results:**

From 2016 to 2022, the incidence rate of PTB in southwest China showed a decreasing trend (AAPC = -10.22, 95% CI: −11.49% ~ −8.94%), while the incidence rate of PTB-DM increased rapidly (AAPC = 14.25, 95% CI: 11.35% ~17.23%). The proportion of PTB-DM among PTB cases increased from 2.96 to 12.28%. PTB-DM patients were characterized by a higher proportion of males and older adult individuals, the age range of the patients was 11 ~ 100 years, with a mean age of 58.21 ± 12.02 years, with multiple positive aetiological results, and lower rates of proactive medical consultation. Among PTB-DM patients, successful outcomes accounted for 80.85%, while unsuccessful outcomes accounted for 19.15%. Although the number of PTB-DM patients with successful treatment outcomes increased overall (AAPC = 12.22, 95% CI: 10.30% ~14.16%), the failure rate showed a gradual upward trend (AAPC = 14.18, 95% CI: 6.53% ~ 27.67%). Older age, retreatment, referral, and multiple positive aetiological results were risk factors for adverse treatment outcomes among PTB-DM patients.

**Conclusion:**

The study presents the true prevalence of PTB-DM comorbidity in the general population of Southwest China, revealing a significant upward trend in its prevalence and a higher risk of adverse outcomes among PTB-DM patients. Future efforts should focus on the prevention and control of PTB-DM comorbidity, with early screening and standardized treatment for high-risk groups such as the older adult, as well as implementing comprehensive and effective treatment and management measures for patients.

## Introduction

Pulmonary tuberculosis (PTB), stemming from *Mycobacterium tuberculosis*, presents as a persistent respiratory infection. Despite global healthcare advancements and the implementation of the United Nations Sustainable Development Goals and the World Health Organization (WHO) End PTB Strategy (1), PTB remains a notable public health concern. According to the latest WHO Global PTB Report for 2023, PTB persists as the second leading cause of death from a single infectious agent globally, trailing only behind COVID-19, with mortality nearly double that of HIV/AIDS. In 2022, there were 10.6 million new PTB cases globally, resulting in 1.3 million deaths (2). Despite efforts, advancements towards eradicating PTB appear daunting, as the cumulative decline in PTB mortalities and incidences from 2015 to 2022 fell markedly below the targeted 50 and 75% reductions by 2025 (3). As a representative high PTB burden country, China has been committed to PTB control efforts, yet it grapples with a substantial burden (4, 5). In 2022, China reported 748,000 new PTB cases, with an incidence rate of 52 /100,000, ranking third among 30 high PTB burden countries worldwide, accounting for 7.1% of global PTB cases (3). The situation for PTB prevention and control in China remains critical.

To achieve the goal of ending PTB and to identify its determinants, the WHO has established a monitoring framework of indicators related to PTB development goals. This framework includes a total of 14 indicators across three areas: socio-economic determinants, universal health coverage and health expenditures, and health-related risk factors (6). Among these health risk factors are malnutrition, HIV infection, alcohol abuse, tobacco use, and diabetes mellitus (DM), with DM being one of the health risk factors (7). Current trends of globalization, aging, and urbanization (migration from rural areas to cities, leading to changes in living standards and dietary patterns) have contributed to a rapid increase in the prevalence and number of DM cases (8). The World Diabetes Report indicates that in 2021, the number of adults with DM globally reached 537 million (1 in 10 individuals having DM), while the number of DM patients in China is 141 million, ranking first in the world (9). The WHO reports DM as the fifth leading risk factor for PTB, attributing 15% of PTB risk factors and 370,000 PTB cases to DM (7). The study by Odone et al. (10) shows that the current upward trend in DM prevalence will counterbalance 3 percent of the decline in global PTB incidence by 2035 and could exacerbate it further in a pessimistic scenario. Numerous clinical medical and immunological studies (11–14) have confirmed that DM increases susceptibility to PTB and the risk of adverse treatment outcomes. The high glucose metabolic state in the tissues of DM patients can lead to tissue hypoxia, resulting in reduced body resistance. Additionally, DM diminishes the ability of macrophages and lymphocytes to present antigens and clear PTB, interfering with the host’s innate and adaptive immune responses, thereby potentially increasing susceptibility to pathogens and worsening disease.

Despite the abundance of clinical and immunological studies on PTB-DM, comprehensive research on the epidemiological trends, treatment outcomes, and risk factors for adverse outcomes related to PTB-DM in specific countries or regions remains limited. A study in the United States (15) reported the prevalence of DM among patients through screening in PTB clinics but did not address the overall epidemiological status or treatment outcomes for the region. Similar screenings for DM among PTB patients have also been reported in India, Sri Lanka, Fiji, and Pakistan (16–19), indicating a high prevalence of DM among PTB patients, but the prevalence of PTB-DM comorbidity in the general population remains unclear. Chongqing, as a municipality and the most populous city in China (32.05 million inhabitants) (20), and is representative of the PTB epidemic and ageing population in the southwest (21). Our study therefore focuses on investigating the comorbidity status of PTB-DM in southwest China by studying Chongqing. Statistical data show that PTB remains the leading notifiable infectious disease in Chongqing, ranking higher than the national average (39.8/100,000) (22). In 2023, people aged 60 and over accounted for 21.87% of Chongqing’s population and a high adult DM prevalence of 14.35% (23). Given the limited reports on the true population-level prevalence of PTB-DM in specific regions and the high risk of PTB-DM comorbidity due to the substantial older adult population and high DM prevalence in Chongqing, we conducted the study on the epidemiological trends, treatment outcomes, and risk factors for adverse outcomes of PTB-DM in Chongqing from 2016 to 2022, with the hope of providing evidence to reduce the risk of PTB-DM comorbidity and improve patient treatment outcomes.

## Methods

### Study design and data sources

**Figure 1:**
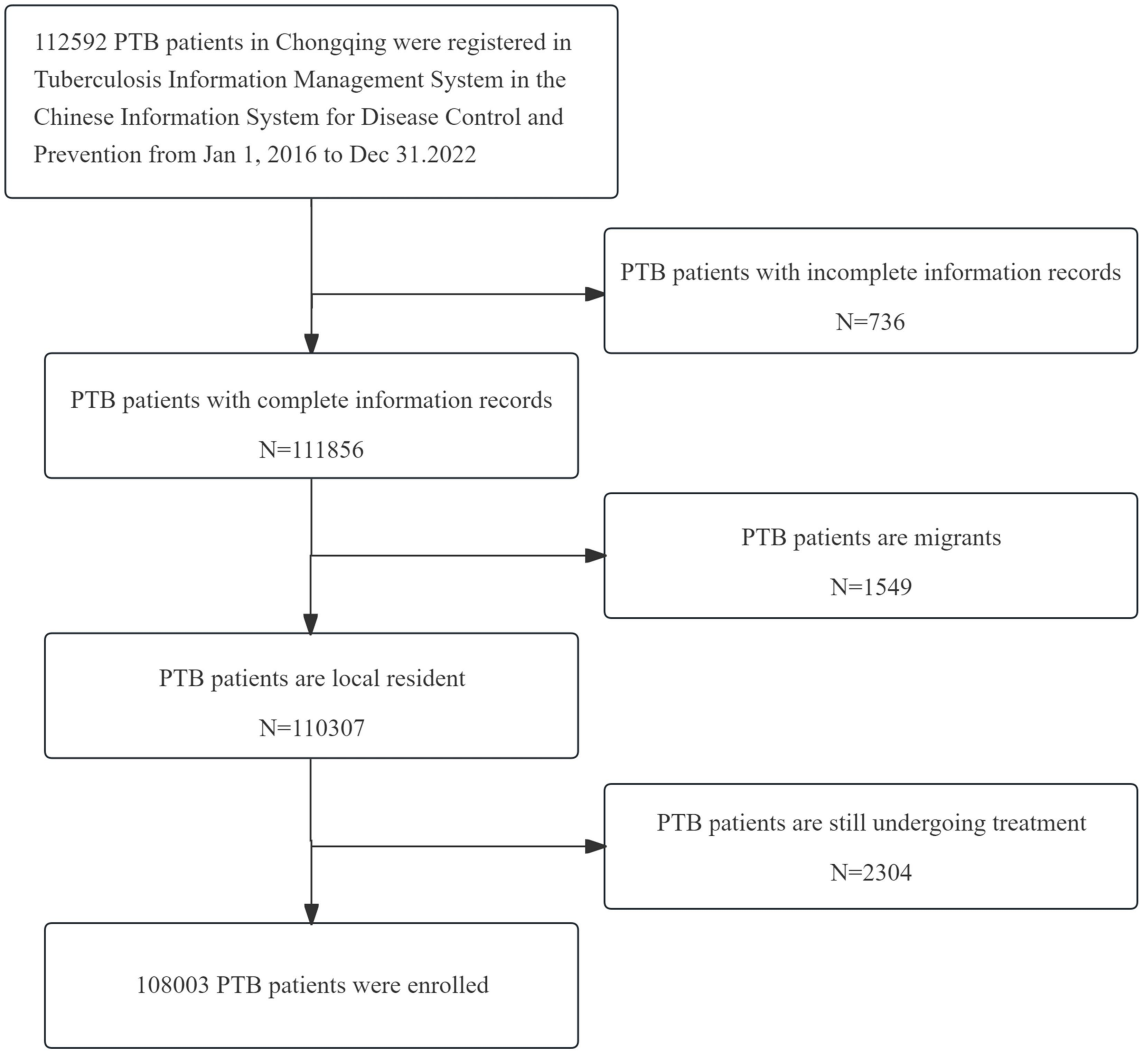
Flow chart of patient inclusion.

The data utilized in this study were sourced from the Tuberculosis Information Management System (TBIMS) within the China Information System for Disease Control and Prevention (CISDP) (24). Patient medical records were collected by the Chongqing Municipal Tuberculosis Prevention and Control Department, then reviewed and reported to the Chinese Center for Disease Control and Prevention. We extracted data from the TBIMS on 112,592 registered PTB patients in Chongqing from 2016 to 2022. After excluding 736 incomplete records, 1,549 patients from non-Chongqing households and 2,304 patients still under treatment without treatment outcome, a total of 108,003 PTB patient records were obtained (Figure 1), with ages ranging from 11 to 100 years. The information collected includes population characteristics such as gender, ethnicity, age, occupation and living region, as well as clinical characteristics such as time of first consultation, source of patients, delayed treatment, aetiological results, comorbidities, treatment history, treatment management models and treatment outcomes. Our study included all regions of Chongqing and all registered PTB patients within the TBIMS. Population data were obtained from the “Chongqing Statistical Yearbook” from 2016 to 2022 (20).

### Definitions

The definitions of clinical characteristics for PTB patients, as well as the diagnoses of DM and PTB-DM, are based on the standards set forth by the WHO tuberculosis guidelines and the Technical Guidelines for Tuberculosis Prevention and Control in China (2, 25), as well as the WHO and Chinese guidelines for the diagnosis of DM (26, 27), as detailed in Table 1.

**Table 1:** Definition of clinical characteristics of PTB patients and diagnostic criteria for DM and PTB-DM.

	Definition and diagnostic criteria
PTB	PTB is defined as any bacteriologically confirmed or clinically diagnosed case of TB involving the lung parenchyma or the tracheobronchial tree.
Aetiological results	This includes positive results, negative results, and no results. Positive aetiological results refer to any of the three methods including sputum smear microscopy, sputum culture, and molecular biology testing yielding a positive result
Treatment history	(1) New patients are those who have never received tuberculosis treatment or have taken anti-tuberculosis drugs for less than one month. (2) Retreated patients are those who have previously received treatment or have taken anti-tuberculosis drugs for more than one month.
Delayed treatment	The interval between the date of the first symptom and the first medical consultation is more than 14 days.
Source of patients	According to the Technical Guidelines for Tuberculosis Prevention and Control in China and the TBIMS, the source of patients is categorized as clinical consultation, recommended for symptoms, referral, follow-up, physical examinations, or other.
Treatment management models	Also based on technical guidelines, treatment management models include full-course management, full-course supervision, supervision in the intensive phase, and others (including self-medication).
Treatment outcomes	1. Successful treatment outcomes include cured and treatment completed. (1) Cured refers to patients completing the prescribed treatment regimen and having at least one negative aetiological result (sputum smear, sputum culture, or molecular biology test) in the last month of treatment. (2) Treatment completed refers to patients completing the prescribed treatment with negative or no aetiological results in the last month of treatment. 2. Unsuccessful treatment outcomes include death, failure, default, transferred to multi-drug resistance treatment, and others. (1) Death refers to patients who died for any reason during treatment, including TB-related and non-TB-related deaths. (2) Failure refers to patients whose aetiological results remain positive after five months of treatment. (3) Default refers to patients who discontinue treatment due to adverse effects. (4) Transferred to multi-drug resistance treatment refers to PTB patients resistant to both isoniazid and rifampicin, requiring additional medications for treatment.
Diagnostic criteria for DM	The diagnosis of DM was based on guidelines established by the WHO (24) and China (25). DM diagnosis should meet one of the following criteria: HbA1C ≥ 6.5% (48 mmol/mol), FPG ≥ 126 mg/dL (7.0 mmol /L), 2hPG ≥ 200 mg/dL (11.1 mmol /L), or already diagnosed as a DM patient.
Diagnostic criteria for PTB-DM	Patients who simultaneously meet the diagnostic criteria for PTB and DM and are registered as PTB combined with DM patients in the TBIMS.

### Statistical analysis

Data analysis was conducted using SPSS 19.0 software. Continuous variables following a normal distribution were represented using mean and standard deviation (SD), while categorical variables such as population characteristics and clinical characteristics were expressed using frequencies (n) and percentages (%). Chi-square tests (expected counts ≥5 in each cell) and Fisher’s exact test (expected counts <5 in any cell) were used for group comparisons of categorical data. Factors influencing patient treatment outcomes with a significance level of *p* < 0.05 in univariate analysis were included in a multivariable logistic regression model. Stepwise backward selection based on the Wald test was used to select variables associated with unsuccessful treatment outcomes, resulting in the final logistic regression model containing all significant variable indicators. Odds ratios (OR) and 95% confidence intervals (95% CI) were calculated in both univariable and multivariable analyses. The validity of the constructed logistic regression model was assessed using the likelihood ratio chi-square test and the coefficient of determination (R^2^). *p* value < 0.05 was considered statistically significant.

Joinpoint 4.9.1.0 software was used to analyze the temporal trends in the number of PTB-DM cases and incidence rate in Chongqing from 2016 to 2022. Joinpoint regression was performed to fit a logarithmic linear model to the incidence rate, with the formula: ln(y) = *α + β x + ϵ*, where y is the incidence rate, *x* is the year, *α* is the constant term, *β* is the regression coefficient, and *ϵ* is the random error term. Trends were expressed as the average annual percent change (AAPC), annual percent change (APC), and their 95% CI. APC reflects changes in different time periods. If APC ≠ AAPC, it indicates different trends in the data set, and when AAPC >0 and 95% CI > 0, it indicates an increasing trend in the incidence rate during that period; when AAPC <0 and 95% CI ≤ 0, it indicates a decreasing trend in the incidence rate; if APC = AAPC, it indicates no joinpoints in the data set and an overall monotonic decreasing or increasing trend (28).

## Results

### General incidence and trend of PTB and PTB-DM

Based on the completeness of case information and inclusion criteria, a total of 108,003 PTB patients with complete data were registered in Chongqing, Southwest China, from 2016 to 2022,with 6,439 cases identified as PTB-DM. The proportion of PTB-DM cases within the PTB cohort increased significantly, from 2.96% in 2016 to 12.28% in 2022, with an average proportion of 6.59%. Concurrently, the average registered incidence rate of PTB was 49.38 /100,000, decreasing from 66.26/100,000 in 2016 to 34.81/100,000 in 2022 (χ^2^ trend = 4.916, *p* < 0.05), with an annual decline rate of 8.79%. Conversely, PTB-DM incidence rate showed a clear upward trend, the average incidence rate of PTB-DM was 2.9/ 100,000, increasing from 1.96 to 4.28 /100,000 (χ2 trend = 4.539, *p* < 0.05), with an annual increase rate of 11.77% (Table 2).

**Table 2:** Incidence of registered PTB and PTB-DM in Chongqing, southwest China, 2016–2022.

Years	Number of population (10,000)	PTB		PTB-DM		
		Number of case	Incidence rate (per 1,000,000)	Number of case	Incidence rate (per 1,000,000)	% PTB-DM among all PTB case
2016	3048.43	20,199	66.26	598	1.96	2.96
2017	3075.16	18,619	60.55	659	2.14	3.54
2018	3101.79	17,744	57.21	806	2.60	4.54
2019	3124.32	16,737	53.57	926	2.96	5.53
2020	3208.93	10,063	31.36	739	2.30	7.34
2021	3212.43	13,456	41.89	1,337	4.16	9.94
2022	3213.34	11,185	34.81	1,374	4.28	12.28
Total	21984.40	108,003	49.38	6,439	2.91	6.59

Joinpoint regression model analysis showed a decline in the registered PTB cases in Chongqing from 2016 to 2022 (AAPC = −9.64, 95% CI: −10. 69% ~ −8.58%, *p* < 0.001; APC = −13.01%, 2016–2020; APC = −2.50%, 2020–2022), accompanied by a decline in incidence rates (AAPC = −10.22, 95% CI: −11.49% ~ −8.94%, *p* < 0.001; APC = −12.34%, 2016–2020; APC = −5.83%, 2020–2022). However, there was an increase in PTB-DM cases from 2016 to 2022 (AAPC = 15.50, 95% CI: 11.75% ~19.38%, *p* < 0.001; APC = 9.55%, 2016–2020; APC = 28. 40%, 2020–2022), accompanied by a rapid increase in incidence rates (AAPC = 14.25, 95% CI: 11.35% ~17.23%, *p* < 0.001; APC = 9.28%, 2016–2020; APC = 24.89%, 2020–2022) (Supplementary Figure S1).

### Population characteristics of PTB-DM patients

**Figure 2:**
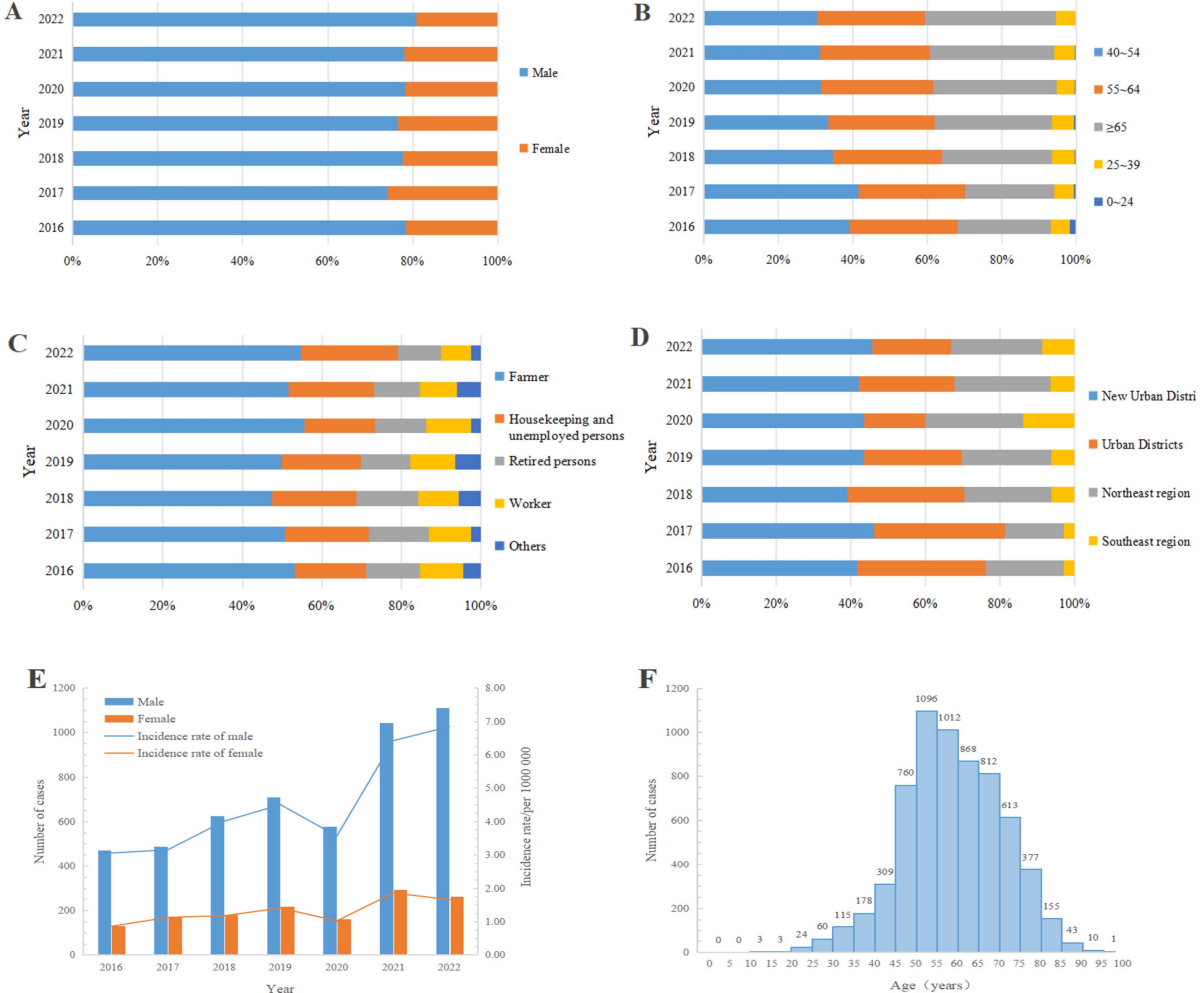
Population characteristics of PTB-DM patients in Chongqing, Southwest China, 2016–2022 (*n* = 6,439). (A) Gender; (B) Age groups; (C) Occupation; (D) Living region; (E) Number of case and incidence rate by gender (reflecting the ratios of the number of male and female cases to their respective populations for each year); (F) Every age.

Analysis of the population characteristics of PTB-DM patients shows that the number of male patients was higher than that of females, with an average male-to-female ratio of 3.51. The average incidence rate for males was 4.50/100,000, while for females it was 1.29/100,000 (Figures 2A,E). The age range of PTB-DM patients spans from 11 ~ 100 years, with a mean age of 58.21 ± 12.02 years. The proportion of patients aged 65 and above is rapidly increasing, with the age groups 40 ~ 54, 55 ~ 64, and ≥ 65 accounting for the vast majority of cases (5,786/6439, 94.05%) (Figures 2B,F). The occupation of the patients was dominated by farmers (3,353/6,439, 52.07%), followed by housekeeping and unemployed persons (1,358/6,439, 21.09%), and retired persons (1,358/6,439, 12.64%) (Figure 2C). In terms of living regions, the New Urban Districts were the most common (2,783/6,439, 43.22%), followed by the Urban Districts (1,686/6,439, 26.18%), and the Northeast Region (1,513/6,439, 23.50%) (Figure 2D).

### Clinical characteristics of PTB-DM patients

**Figure 3:**
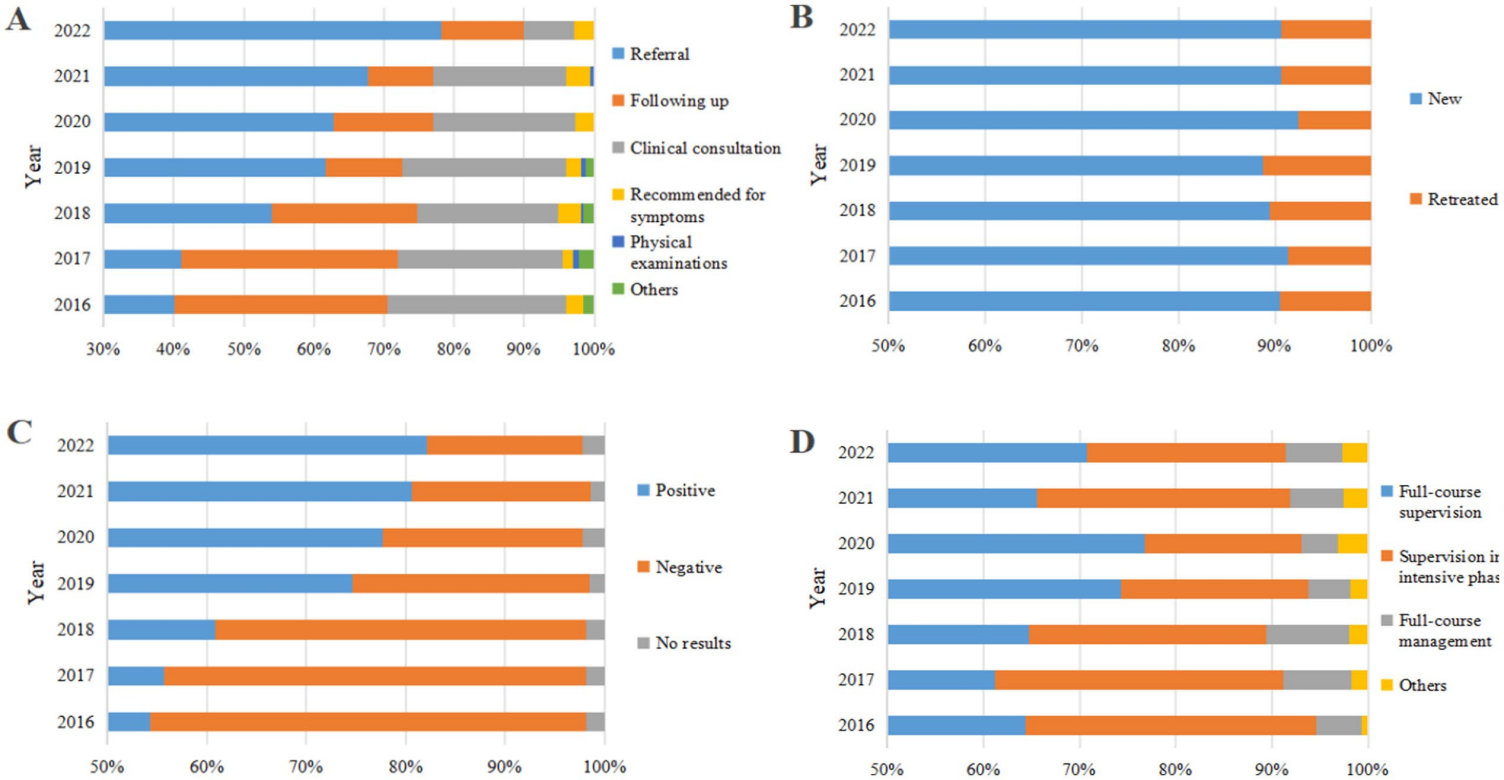
Clinical characteristics of PTB-DM patients in Chongqing, Southwest China, 2016–2022 (*n* = 6,439). (A) Source of patients; (B) Treatment history; (C) Aetiological results; (D) Treatment management models.

Analyses of the clinical characteristics of PTB-DM patients showed some changes in their treatment characteristics. In the source of patients, the proportion of patients following up and clinical consultations decreased, while the proportion of referrals markedly increased to 78.09% (Figure 3A). Regarding treatment history, new patients predominated (5,825/6439, 90.46%), and retreated patients had a minor proportion (614/6439, 9.54%) (Figure 3B). For patient aetiological results, the proportion of positive outcomes is increasing rapidly, from 54.35 to 82.10% (Figure 3C). In the treatment management model, the proportion of full-course supervision initially increased and then decreased, while supervision in the intensive phase initially decreased and then increased, and other management modes remained unchanged (Figure 3D).

### Comparison of PTB and PTB-DM patient characteristics

Analysis of the characteristics within the groups of patients with PTB without DM and those with PTB-DM and showed that PTB-DM patients had higher proportions of male (78.02% vs. 70.71%) and Han ethnicity (95.95% vs. 89.47%), as well as in age groups 40 ~ 54 years, 55 ~ 64 years, and ≥ 65 years (94.05% vs. 66.65%), and positive aetiological results (72.25% vs. 43.09%). Conversely, the proportion of clinical consultation was lower among PTB-DM (18.47% vs. 26.74%) (*p* < 0.001). Additionally, the treatment success rate among PTB-DM patients was lower at 80.85% compared to 88.11% among PTB patients without DM, indicating a notable disparity in treatment success rates between the two cohorts(*p* < 0.001). No differences were observed in the characteristics of local registered residence and delayed treatment between PTB without DM and PTB-DM patients (Table 3).

**Table 3:** Population and clinical characteristics of PTB without DM and PTB-DM patients in Chongqing, Southwest China, 2016–2022 (*n* = 108,003).

Variables	Total (*n* = 108,003)	PTB-DM (*n* = 6,439) No. (%)	PTB without DM (*n* = 101,564) No. (%)	*χ2*	*P* value
Gender				157.808	<0.001
Male	76,840 (71.15)	5,024 (78.02)	71,816 (70.71)		
Female	31,163 (28.85)	1,415 (21.98)	29,748 (29.29)		
Ethnicity				278.609	<0.001
Han	97,048 (89.86)	6,178 (95.95)	90,870 (89.47)		
Minority	10,955 (10.14)	261 (4.05)	10,694 (10.53)		
Age (years)				2351.336	<0.001
≥65	29,047 (26.89)	2011 (31.23)	27,036 (26.62)		
0 ~ 24	17,739 (16.42)	30 (0.47)	17,709 (17.44)		
25 ~ 39	16,520 (15.30)	353 (5.48)	16,167 (15.92)		
40 ~ 54	25,862 (23.95)	2,165 (33.62)	23,697 (23.33)		
55 ~ 64	18,835 (17.44)	1,880 (29.20)	16,955 (16.69)		
Occupation				1650.639	<0.001
Farmer	60,481 (56.00)	3,353 (52.07)	57,128 (56.25)		
Worker	20,658 (19.13)	639 (9.92)	20,019 (19.71)		
Housekeeping and unemployed persons	18,160 (16.81)	1,358 (21.09)	16,802 (16.54)		
Retired persons	4,365 (4.04)	814 (12.64)	3,551 (3.50)		
Others	4,339 (4.02)	275 (4.27)	4,064 (4.00)		
Local registered residence				2.378	0.123
Yes	7,522 (6.96)	479 (7.44)	7,043 (6.93)		
No	100,481 (93.04)	5,960 (92.56)	94,521 (93.07)		
Living region				1436.500	<0.001
Urban Districts	19,475 (18.03)	1,686 (26.18)	17,789 (17.52)		
New Urban Districts	31,392 (29.07)	2,783 (43.22)	28,609 (28.17)		
Northeast region	37,447 (34.67)	1,513 (23.50)	35,934 (35.38)		
Southeast region	19,689 (18.23)	457 (7.10)	19,232 (18.94)		
Source of patients				427.699	<0.001
Clinical consultation	28,344 (26.24)	1,189 (18.47)	27,155 (26.74)		
Recommended for symptoms	2,152 (1.99)	173 (2.69)	1,979 (1.95)		
Referral	54,852 (50.79)	3,960 (61.50)	50,892 (50.11)		
Following up	20,158 (18.66)	1,046 (16.24)	19,112 (18.82)		
Physical examinations	1,862 (1.72)	23 (0.36)	1,839 (1.81)		
Others	635 (0.59)	48 (0.75)	587 (0.58)		
Treatment history				84.227	<0.001
Retreated	95,499 (88.42)	614 (9.54)	6,679 (6.58)		
New	12,504 (11.58)	5,825 (90.46)	94,885 (93.42)		
Aetiological results				2087.056	<0.001
Positive	48,419 (44.83)	4,652 (72.25)	43,767 (43.09)		
Negative	54,289 (50.27)	1,669 (25.92)	52,620 (51.81)		
No results	5,295 (4.90)	118 (1.83)	5,177 (5.10)		
Delayed treatment				0.150	0.698
Yes	76,491 (70.82)	4,574 (71.04)	71,917 (70.81)		
No	31,512 (29.18)	1865 (28.96)	29,647 (29.19)		
Treatment management models				918.897	<0.001
Full-course management	11,169 (10.34)	369 (5.73)	10,800 (10.63)		
Full-course supervision	39,339 (36.42)	4,413 (68.54)	49,926 (49.16)		
Supervision in intensive phase	38,117 (35.29)	1,515 (23.53)	36,602 (36.04)		
Others	19,378 (17.94)	142 (2.21)	4,236 (4.17)		
Treatment outcomes				295.005	<0.001
Successful	94,691 (87.67)	5,206 (80.85)	89,485 (88.11)		
Unsuccessful	13,312 (12.33)	1,233 (19.15)	12,079 (11.89)		

### Treatment outcomes of PTB-DM patients

**Figure 4:**
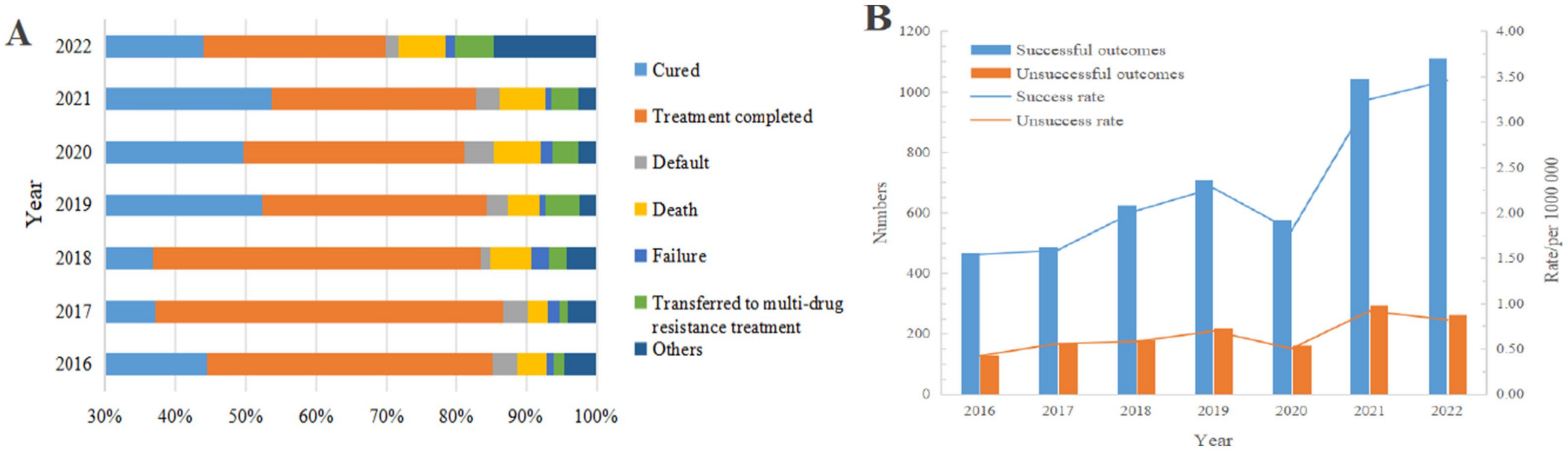
Treatment outcomes of PTB-DM patients. (A) Treatment outcomes; (B) Number and rate of successful and unsuccessful treatment outcomes (reflecting the ratios of successful and unsuccessful cases to the total population for each year).

Among patients registered with PTB-DM in Chongqing, the treatment outcomes were as follows: successful outcomes accounted for 80.85% (5,206/6439), while unsuccessful outcomes accounted for 19.15% (1,233/6439). Among those with successful outcomes, there were 2,980 cured (46.28%), and 2,226 completed treatment (34.57%). Among those with unsuccessful treatment, there were 363 deaths (5.64%), 180 defaults (2.80%), 88 failure (1.37%), 235 transferred to multi-drug resistance treatment (3.65%), and 367 others (5.70%). The average successful treatment rate for PTB-DM patients from 2016 to 2022 was 82.01%, with an annual decrease rate of 2.81% (Table 4, Figure 4).

**Table 4:** Treatment outcomes of PTB-DM patients registered for treatment in Chongqing, Southwest China, 2016–2022 (*n* = 6,439).

Years	Successful				Unsuccessful						
	Cured	Treatment completed	Total	Percentage (%)	Default	Death	Failure	Transferred to multi-drug resistance treatment	Others	Total	Percentage (%)
2016 (*n* = 598)	266	244	510	85.28	21	25	6	8	28	88	14.72
2017 (*n* = 659)	245	327	572	86.80	23	18	11	8	27	87	13.20
2018 (*n* = 806)	296	377	673	83.50	11	47	20	20	35	133	16.50
2019 (*n* = 926)	484	298	782	84.45	27	42	8	44	23	144	15.55
2020 (*n* = 739)	367	233	600	81.19	31	49	13	27	19	139	18.81
2021 (*n* = 1,337)	717	392	1,109	82.95	43	87	11	52	35	228	17.05
2022 (*n* = 1,374)	605	355	960	69.87	24	95	19	76	200	414	30.13
Total	2,980	2,226	5,206	80.85	180	363	88	235	367	1,233	19.15

Joinpoint regression model analysis showed that although the total number of PTB-DM patients successfully treated increased from 2016 to 2022 (AAPC = 12.22,95%CI: 10.30% ~ 14.16%,*p* < 0.001;APC = 8.88%, 2016–2020; APC = 19.20%, 2020–2022) (Figure 4), the proportion of successful treatments decreased from 85.28 to 82.95% (AAPC = -2.15, 95% CI: −4.01% ~ 0.10%, *p* < 0.001). In addition, the number of patients with unsuccessful treatments gradually increased (AAPC = 26.66, 95% CI: 13.78% ~ 40.99%, *p* < 0.001), and the proportion of unsuccessful treatments increased from 14.72 to 17.05% (AAPC = 14.18,95%CI: 6.53% ~ 27.67%, *p* < 0.001) (Supplementary Figure S2).

### Characteristics of PTB-DM patients with different treatment outcomes

Analysis of the characteristic of PTB-DM patients across different treatment outcomes showed that, compared to patients with successful treatment outcomes, those with unsuccessful outcomes displayed a higher proportion of males (81.27% vs. 77.26%), as well as in age groups 40 ~ 54 years, 55 ~ 64 years, and ≥ 65 years (95.3% vs. 93.76%), and retreated patients (13.06% vs. 8.70%), along with positive aetiological results (80.54% vs. 70.28%) (*p* < 0.001). There were no differences between patients with different treatment outcomes in terms of local registered residence (Table 5).

**Table 5:** Characteristics of PTB-DM patients with successful and unsuccessful treatment outcomes in Chongqing, Southwest China, 2016–2022 (*n* = 6,439).

Variables	Total (*n* = 6,439)	Successful (*n* = 5,206) No. (%)	Unsuccessful (*n* = 1,233) No. (%)	*χ2/ Z*	*P* value
Gender				9.341^a^	<0.001
Male	5,024 (78.02)	4,022 (77.26)	1,002 (81.27)		
Female	1,415 (21.98)	1,184 (22.74)	231 (18.73)		
Ethnicity				5.820^a^	0.016
Han	6,178 (95.95)	5,010 (96.24)	1,168 (94.73)		
Minority	261 (4.05)	196 (3.76)	65 (5.27)		
Age (years)				86.328^b^	<0.001
≥65	2,011 (31.23)	1,505 (28.91)	506 (41.04)		
0 ~ 24	30 (0.47)	28 (0.54)	2 (0.16)		
25 ~ 39	353 (5.48)	297 (5.70)	56 (4.54)		
40 ~ 54	2,165 (33.62)	1,857 (35.67)	308 (24.98)		
55 ~ 64	1,880 (29.20)	1,519 (29.18)	361 (29.28)		
Occupation				17.418^a^	0.002
Farmer	3,353 (52.07)	2,701 (51.88)	652 (52.88)		
Worker	639 (9.92)	548 (10.53)	91 (7.38)		
Housekeeping and unemployed persons	1,358 (21.09)	1,101 (21.15)	257 (20.84)		
Retired persons	814 (12.64)	629 (12.08)	185 (15.00)		
Others	275 (4.27)	227 (4.36)	48 (3.89)		
Local registered residence				1.109^a^	0.292
Yes	479 (7.44)	396 (7.61)	83 (6.73)		
No	5,960 (92.56)	4,810 (92.39)	1,150 (93.27)		
Living region				17.016^a^	0.001
Urban Districts	1,686 (26.18)	1,353 (25.99)	333 (27.01)		
New Urban Districts	2,783 (43.22)	2,277 (43.74)	506 (41.04)		
Northeast region	1,513 (23.50)	1,238 (23.78)	275 (22.30)		
Southeast region	457 (7.10)	338 (6.49)	119 (9.65)		
Source of patients				20.163^b^	0.001
Clinical consultation	1,189 (18.47)	995 (19.11)	194 (15.73)		
Recommended for symptoms	173 (2.69)	145 (2.79)	28 (2.27)		
Referral	3,960 (61.50)	3,135 (60.22)	825 (66.91)		
Following up	1,046 (16.24)	869 (16.69)	177 (14.36)		
Physical examinations	23 (0.36)	19 (0.36)	4 (0.32)		
Others	48 (0.75)	43 (0.83)	5 (0.41)		
Treatment history				21.929^a^	<0.001
Retreated	614 (9.54)	453 (8.70)	161 (13.06)		
New	5,825 (90.46)	4,753 (91.30)	1,072 (86.94)		
Aetiological results				57.155^a^	<0.001
Positive	4,652 (72.25)	3,659 (70.28)	993 (80.54)		
Negative	1,669 (25.92)	1,454 (27.93)	215 (17.44)		
No results	118 (1.83)	93 (1.79)	25 (2.03)		
Delayed treatment				409.663^a^	<0.001
Yes	4,574 (71.04)	3,988 (76.60)	586 (47.53)		
No	1,865 (28.96)	1,218 (23.40)	647 (52.47)		
Treatment management models				204.337^a^	<0.001
Full-course management	369 (5.73)	203 (3.90)	166 (13.46)		
Full-course supervision	4,413 (68.54)	3,683 (70.75)	730 (59.21)		
Supervision in intensive phase	1,515 (23.53)	1,229 (23.61)	286 (23.20)		
Others	142 (2.21)	91 (1.75)	51 (4.14)		

a For results of chi-square test.

b For results of Fisher’s exact test.

### Risk factors analysis for unsuccessful treatment outcomes of PTB-DM patients

We performed univariate and multivariate analyses on the population and clinical characteristics of PTB-DM patients to explore the risk factors associated with unsuccessful treatment outcomes. In the univariate analysis, factors associated with higher treatment success rates included minority (OR = 1.277, 95% CI: 1.091 ~ 1.494, *p* < 0.001); age group of 0 ~ 24 years (OR = 4.707, 95% CI: 1.117 ~ 19.829, *p* = 0.035), 25 ~ 39 years (OR = 1.783, 95%CI: 1.317 ~ 2.414, *p* < 0.001), 40 ~ 54 years old (OR = 2.027, 95%CI: 1.732 ~ 2.372, *p* < 0.001), 55 ~ 64 years old (OR=2.415, 95%CI: 1.214~1.648, *p* < 0.001); being workers (OR = 1.475, 95%CI: 1.162 ~ 1.873, *p* < 0.001); being new patients (OR = 1.576, 95%CI: 1.301 ~ 1.909, *p* < 0.001);and negative aetiological results (OR = 1.835, 95% CI: 1.565 ~ 2.153, *p* < 0.001). Conversely, characteristics associated with lower treatment success rates included female gender (OR = 0.703, 95%CI: 0.527 ~ 0.937, *p* < 0.05); being retired persons (OR = 0.822, 95%CI: 0.684 ~ 0.989, *p* < 0.05); residing in the southeast region (OR = 0.649, 95%CI: 0.543 ~ 0.880, *p* < 0.05); and being referred patients (OR = 0.741, 95%CI: 0.624 ~ 0.880, *p* < 0.05).

Characteristics with a significance level of *p* < 0.05 from the univariate analysis were included in the multivariate logistic regression analysis. The multivariate analysis identified Han ethnicity, age ≥ 65 years, residing in southeast region, being referred patients, being retreated patients, and positive aetiological results as significant risk factors for unsuccessful treatment outcomes among PTB-DM patients, while patients with other characteristics have higher treatment success rates. Specifically, factors associated with higher treatment success rates included minority (OR = 1.359, 95%CI: 1.155 ~ 1.598, *p* < 0.001); age groups of 0 ~ 24 years (OR = 4.582, 95%CI: 1.072 ~ 19.593, *p* = 0.040), 25 ~ 39 years (OR = 1.802, 95%CI: 1.312 ~ 2.475, *p* < 0.001), 40 ~ 54 years (OR = 2.005, 95%CI: 1.693 ~ 2.374, *p* < 0.001), and 55 ~ 64 years (OR = 1.399, 95%CI: 1.195 ~ 1.638, *p* < 0.001); being new patients (OR = 1.540, 95%CI: 1.262–1.879, *p* < 0.001); and negative aetiological results (OR = 1.731, 95%CI: 1.471 ~ 2.037, *p* < 0.001). Conversely, factors associated with lower treatment success rates included residing in the Southeast region (OR = 0.621, 95%CI: 0.442 ~ 0.872, *p* = 0.006), and being referred patients (OR = 0.778, 95%CI: 0.651 ~ 0.930, *p* < 0.05) (Table 6). The constructed logistic regression model yielded a log-likelihood of 4078.649 and with R^2^ = 0.52 (*p* < 0.001), indicating statistical significance.

**Table 6:** Univariable and multivariable analysis for unsuccessful treatment outcomes of PTB-DM patients.

Variables	Univariable		Multivariable	
	OR (95% CI)	*P* value	OR (95% CI)	*P* value
Gender				
Male	Reference	Reference	Reference	Reference
Female	1.113 (0.562 ~ 1.237)	0.002	1.029 (0.681 ~ 1.554)	0.892
Ethnicity				
Han	Reference	Reference	Reference	Reference
Minority	1.277 (1.091 ~ 1.494)	< 0.001	1.359 (1.155 ~ 1.598)	< 0.001
Age (years)				
≥65	Reference	Reference	Reference	Reference
0 ~ 24	4.707 (1.117 ~ 19.829)	0.035	4.582 (1.072 ~ 19.593)	0.040
25 ~ 39	1.783 (1.317 ~ 2.414)	< 0.001	1.802 (1.312 ~ 2.475)	< 0.001
40 ~ 54	2.0.027 (1.732 ~ 2.372)	< 0.001	2.005 (1.693 ~ 2.374)	< 0.001
55 ~ 64	2.415 (1.214 ~ 1.648)	< 0.001	1.399 (1.195 ~ 1.638)	< 0.001
Occupation				
Farmer	Reference	Reference	Reference	Reference
Worker	1.475 (1.162 ~ 1.873)	< 0.001	1.166 (0.905 ~ 1.503)	0.234
Housekeeping and unemployed persons	1.036 (0.883 ~ 1.216)	0.665	0.935 (0.784 ~ 1.116)	0.458
Retired persons	0.822 (0.684 ~ 0.989)	0.038	0.968 (0.7911.184~)	0.749
Others	1.144 (0.828 ~ 1.580)	0.415	1.028 (0.734 ~ 1.440)	0.871
Local registered residence				
Yes	Reference	Reference		
No	0.877 (0.686 ~ 1.120)	0.293		
Living region				
Urban Districts	Reference	Reference	Reference	Reference
New Urban Districts	1.095 (0.939 ~ 1.278)	0.248	1.077 (0.911 ~ 1.273)	0.384
Northeast region	1.081 (0.906 ~ 1.291)	0.388	1.062 (0.872 ~ 1.293)	0.549
Southeast region	0.649 (0.543 ~ 0.880)	0.003	0.621 (0.442 ~ 0.872)	0.006
Source of patients				
Clinical consultation	Reference	Reference	Reference	Reference
Recommended for symptoms	1.010 (0.655 ~ 1.557)	0.965	1.008 (0.642 ~ 1.582)	0.973
Referral	0.741 (0.624 ~ 0.880)	0.001	0.778 (0.651 ~ 0.930)	0.006
Following up	0.957 (0.766 ~ 1.197)	0.701	0.958 (0.761 ~ 1.206)	0.715
Physical examinations	0.926 (0.312 ~ 2.752)	0.890	0.852 (0.280 ~ 2.587)	0.777
Others	1.677 (0.656 ~ 4.287)	0.281	1.540 (1.262 ~ 1.879)	0.157
Treatment history				
Retreated	Reference	Reference	Reference	Reference
New	1.576 (1.301 ~ 1.909)	< 0.001	1.540 (1.262 ~ 1.879)	< 0.001
Aetiological results				
Positive	Reference	Reference	Reference	Reference
Negative	1.835 (1.565 ~ 2.153)	< 0.001	1.731 (1.471 ~ 2.037)	< 0.001
No results	1.010 (0.646 ~ 1.579)	0.967	1.080 (0.686 ~ 1.701)	0.739
Delayed treatment				
Yes	Reference	Reference		
No	1.025 (0.894 ~ 1.176)	0.720		
Treatment management models				
Full-course management	Reference	Reference		
Full-course supervision	0.894 (0.521 ~ 1.533)	0.683		
Supervision in intensive phase	0.702 (0.439 ~ 1.122)	0.139		
Others	0.776 (0.479 ~ 1.255)	0.301		

## Discussion

In this study, we investigated the prevalence trend of PTB-DM registered in TBIMS within the CISDP in Chongqing. The results indicate a downward trend in the incidence of PTB (AAPC = −9.64, 95% CI: −10.69% to −8.58%), suggesting certain achievements in PTB control efforts in Chongqing, consistent with the overall declining trend in PTB incidence in China (5, 29). However, there is a clear upward trend in the incidence of PTB-DM (AAPC = 14.38, 95% CI: 7.08% ~23.73%), with the incidence rising from 1.96/100,000 to 4.28/100,000. In our study, the prevalence of DM among PTB patients (the ratio of PTB-DM to all PTB cases) is 12.28%, which is higher than the DM prevalence of 11.6% in the general population of Chongqing that year, consistent with numerous research findings (30, 31). A study in South Asia showed that the prevalence of DM among PTB patients in the region was 21%, ranging from 11% in Bangladesh to 24% in Sri Lanka, and that the prevalence would be higher in those countries with a high burden of PTB, and that these figures are significantly higher compared with the global DM prevalence rate of 15.4%(95%CI: 14.1% ~ 16.6%) and the Asian DM prevalence rate of 17%(95%CI: 11.4% ~ 25.8%) (32). Furthermore, large-scale DM screening among PTB patients in China also revealed higher DM prevalence rates (29). This suggests that bidirectional screening for PTB and DM has a scientific basis and can expedite the identification of susceptible individuals and potential cases. Although the WHO and the International Union Against Tuberculosis and Lung Disease proposed the Collaborative Framework for Care and Control of Tuberculosis and Diabetes in 2011 (33), many countries currently perform more DM screening among PTB patients than PTB screening among DM patients (34). Given the increasingly urgent comorbidity trend of PTB-DM, bidirectional screening for PTB and DM should be on the agenda and implemented promptly.

Analysis of the population and clinical characteristics of PTB-DM patients in Chongqing, southwestern China, revealed a higher proportion of Male, predominantly middle-aged and older adults, and more positive aetiological results in PTB-DM patients. In our study, the male-to-female ratio of affected individuals averaged 3.51, with a mean age of 58.21 ± 12.02 years, and the middle-aged and older adult age group accounted for over 90% of the patient population. The higher prevalence of PTB-DM among males and the older adult, which is consistent with previous studies (35–37), may be attributed to several factors. First, males are more likely to engage in outdoor employment and social activities, higher smoking rates, and lower awareness of personal hygiene practices, such as mask-wearing, compared to females (4, 38), thereby increasing the risk of PTB exposure. Second, males typically have diets high in fats and sugars, while females tend to control their diets and focus on exercise (30), contributing to higher DM prevalence among males. Additionally, the immune status of DM patients and their medication regimens may further elevate the risk of PTB (39, 40). Collectively, these factors contribute to a higher PTB-DM comorbidity risk in males. Furthermore, the older adult encounter a decreased basal metabolic rate, increased secretion of *β*-cell and insulin, resulting in diminished compensatory function against glucose stimulation, thereby elevating the risk of positive aetiological results and comorbidity among this demographic (41, 42). Moreover, PTB-DM patients were more commonly identified through referrals or follow-ups rather than seeking clinical consultation, which mirroring findings in related reports (37). This may be influenced by China’s diagnostic and management systems for PTB and the lifestyle patterns of the older adult population. Older adult individuals, residing alone and grappling with mobility constraints, coupled with the presence of multiple chronic diseases, may present atypical clinical manifestations post-PTB, consequently resulting in fewer proactive medical visits by older adult patients (42). Furthermore, the Technical Guidelines for Tuberculosis Prevention and Control in China mandate timely reporting and referral of diagnosed PTB patients to designated hospitals, rendering referral and follow-up the principal avenues for patient identification (24, 25). Our findings showed that over half of PTB-DM patients were farmers, aligning with findings from Ling et al. (36), Jiang et al. (43), and Tusun et al. (44). Given the generally low level of healthcare services in rural areas (45), this highlights the need to strengthen the implementation of rural public health services and enhance the accessibility of prevention and treatment interventions. Additionally, the overwhelming majority of PTB-DM patients are new cases (90.46%), while the number of retreated patients is minimal, possibly due to the gradual establishment of patient diagnosis and management measures in China, resulting in a decreasing trend in retreated patient numbers.

Our investigation of treatment outcomes in PTB-DM patients compared to PTB patients showed that DM comorbidity increased the risk of unsuccessful treatment outcomes. The treatment success rate of PTB-DM patients was 80.85%, lower than the treatment success rate of 88.11% for PTB patients alone and notably lower than the summarized treatment success rate of 93.9% for new PTB cases in mainland China (46), which is consistent with the results of other studies (47, 48). Joinpoint regression analysis of treatment outcomes for PTB-DM patients revealed a general increase in the number of successfully treated patients, but this was accompanied by a gradual increase in treatment failure rates (AAPC = 14.18, 95% CI: 6.53% ~ 27.67%), indicating a less optimistic prognosis for patient outcomes. Studies suggest that anti-TB medications may exacerbate complications of DM itself, thereby increasing the risk of adverse reactions or mortality (49, 50). Furthermore, owing to the prolonged treatment duration and multiple comorbidities in these patients, their motivation to adhere to standardized treatment regimens is diminished, with issues such as failing to take medication on time or forgetting to take medication (30, 42). This highlights the need to strengthen disease screening and health management for PTB-DM comorbid patients, along with implementing patient tracking and disease assessment to ensure they receive more effective and sustained treatment.

Analysis of risk factors for unsuccessful treatment outcomes among patients indicates that, in univariable analysis, besides population characteristics such as gender and age, factors related to the source of patients, treatment history, and treatment management models also influence the outcomes of PTB-DM patients. This underscoring the proactive role of timely treatment and effective management in the prevention and control of PTB-DM, suggesting the need for more efficient and comprehensive patient management strategies. Multivariable analysis results revealed that advanced age is a risk factor for the treatment outcomes of PTB-DM, with the cure rate gradually decreasing as age increases among the middle-aged and older adult population, consistent with previous research findings (42, 49). Etiological research (7, 32, 51) indicates that elevated glucose levels in the blood and tissues of DM patients provide a nutrient-rich environment for the growth and reproduction of mycobacterium tuberculosis. Furthermore, increased levels of glucuronic acid create an acidic environment that reduces antibody levels; a high-glucose metabolic state results in tissue hypoxia and decreased body resistance, leading to diminished immune function and increased susceptibility. Additionally, due to decreased pulmonary function and blood flow in the lungs, slower lesion absorption in the older adult may also affect drug absorption (52). In our study, the cure rate among ethnic minorities was higher than that among the Han ethnic group, which differs from other studies that showed no racial differences in treatment outcomes (53) or reported higher success rates among Han patients (54). The higher cure success rate among ethnic minorities in our study may be due to the smaller number of patients. Research results revealed that the treatment success rate was higher among newly treated patients and those with negative aetiological results, being 1.540 times (95% CI: 1.262 ~ 1.879) and 1.731 times (95% CI: 1.471 ~ 2.037) higher than that among retreated patients and those with positive aetiological results, respectively, consistent with related studies in China, the USA, and Europe (15, 55, 56). Patients with positive aetiological results and those who are retreated generally have poorer treatment outcomes due to longer treatment times, higher bacterial loads, severe immune damage, and higher disease severity. The results also showed that the success rate of referred patients is lower than that of symptomatic patients seeking treatment (OR = 0.782, 95% CI: 0.654 ~ 0.935), which is consistent with the findings of Wu and Mave (30, 53). The poorer outcomes for referred patients may be because suspected patients do not seek initial treatment at designated TB medical institutions after symptom onset, leading to delayed treatment and intervention during the referral process, thereby exacerbating the disease (57, 58). Additionally, the cure rate was lower in the southeast of Chongqing (OR = 0.621, 95%CI: 0.442 ~ 0.872), consistent with research conclusions on the incidence and spatial distribution characteristics of PTB in Chongqing, indicating that the southeastern region is a hotspot and high clustering area for PTB incidence (47), highlighting the need to strengthen prevention and control efforts in this region to reduce the incidence rate.

Our study has several limitations. Firstly, we investigated the comorbidity status of PTB-DM in southwest China by studying Chongqing, but we did not directly study the overall prevalence data and situation in southwest China, which is not fully representative of the comorbidity trends in southwest China. Secondly, although we obtained case information on PTB-DM patients from DCPIS, certain clinical details such as levels of blood glucose and lipids were unavailable, hindering analysis of risk factors and high-risk populations for PTB-DM comorbidity. Thirdly, our data originated from case information registered in TBIMS within the CISDP. While this system covers the majority of PTB patients, there may still be undetected or untreated cases, potentially resulting in an underestimation of the true prevalence to some extent.

## Conclusion

This study investigated the epidemiological trends, treatment outcomes, and risk factors for adverse outcomes of PTB-DM in the whole population based on surveillance data in Chongqing, Southwest China. It addresses the gap in previous PTB-DM research, which primarily focused on the immunological relationship between DM and PTB, lacking reports on the actual prevalence of PTB-DM comorbidity. The findings indicate a significant upward trend in the prevalence of PTB-DM in the southwestern region of China, which is characterized by a high proportion of older adult patients, numerous positive aetiological results, and low rates of proactive medical consultation. Treatment and management factors significantly influence the outcomes for PTB-DM patients, highlighting the need for early screening and standardized treatment to improve patient management and treatment outcomes. Furthermore, future research should incorporate more specific clinical indicators, as patients’ own health metrics have a substantial clinical impact on treatment outcomes, in addition to management factors. Additionally, while current studies indicate a higher prevalence of PTB among DM patients, the necessity and benefits of conducting PTB screening for all DM patients warrant further research and evaluation.

## Data Availability

The original contributions presented in the study are included in the article/Supplementary material, further inquiries can be directed to the corresponding authors.
