# Supplementary material for: Prevalence trends, population characteristics and treatment outcomes of tuberculosis combined with diabetes in Southwest China: a register-based retrospective study
Source: Front Public Health. 2024 Nov 20;12:1445857. doi: 10.3389/fpubh.2024.1445857 (PMC11616032; doi:10.3389/fpubh.2024.1445857)
Supplement: Supplementary file 1 [file Data_Sheet_1.docx]

***Supplementary Material***

**Supplementary Figure 1.**Incidence and trend of PTB and PTB-DM in Chongqing ,southwest China, 2016–2022.

**Supplementary Figure 2.**Number and trends of successful and unsuccessful treatment outcomes of PTB-DM patients registered for treatment in Chongqing ,southwest China, 2016–2022.

| 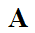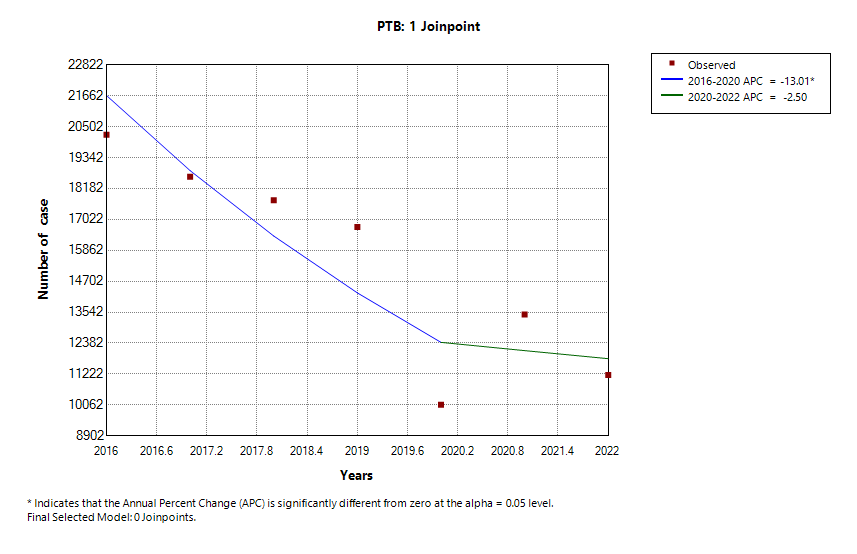 | 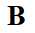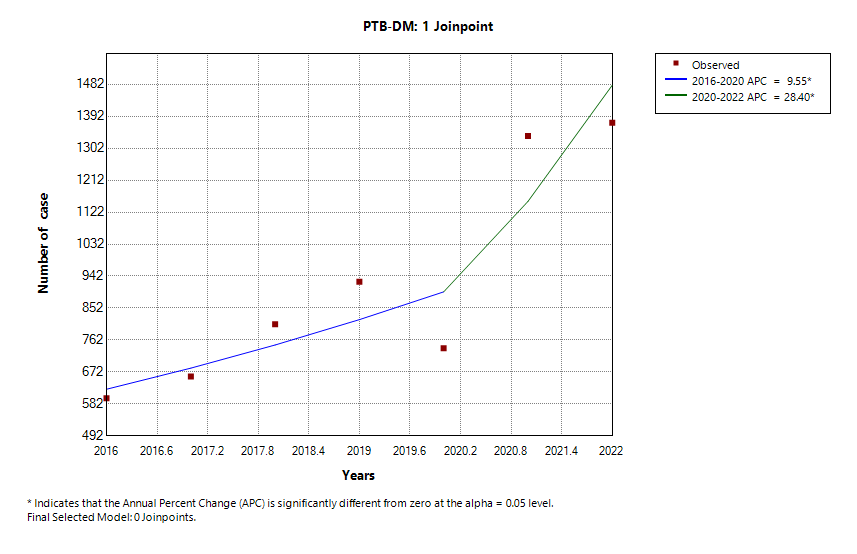 |
| --- | --- |
| 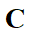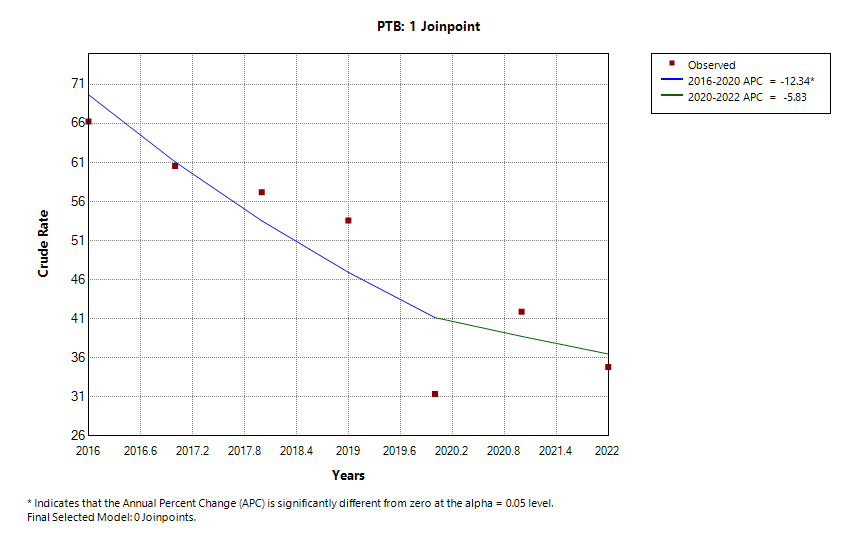 | 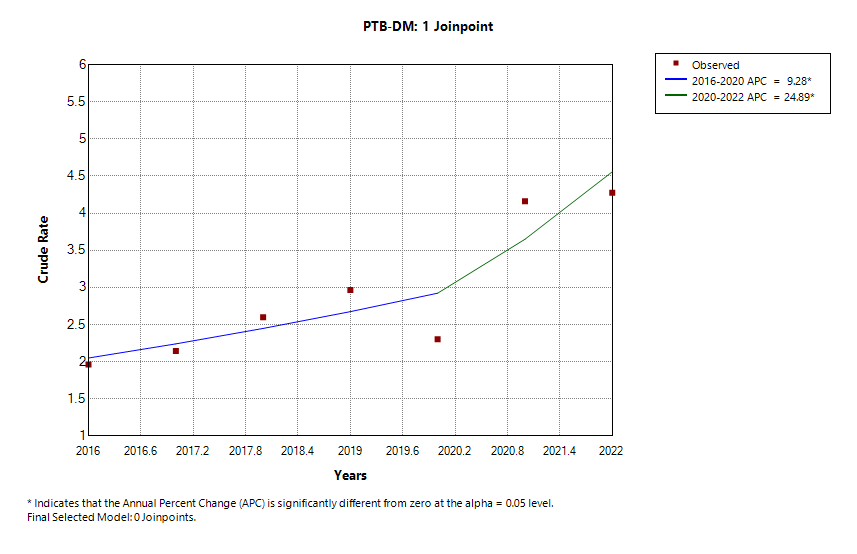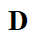 |

**Supplementary Figure 1.**Incidence and trend of PTB and PTB-DM in Chongqing ,southwest China, 2016–2022.(A) Number of cases of PTB. (B)Number of cases of PTB-DM. (C)Crude incidence rate of PTB. (D)Crude incidence rate of PTB-DM.

*Indicate that the Annual Percent Change (APC) is significantly different from zero at the alpha = 0.05 level .

| 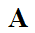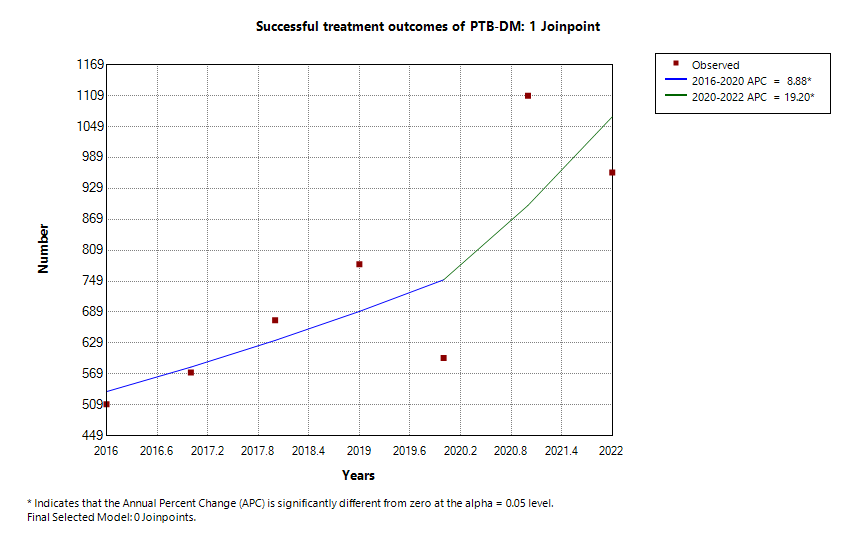 | 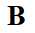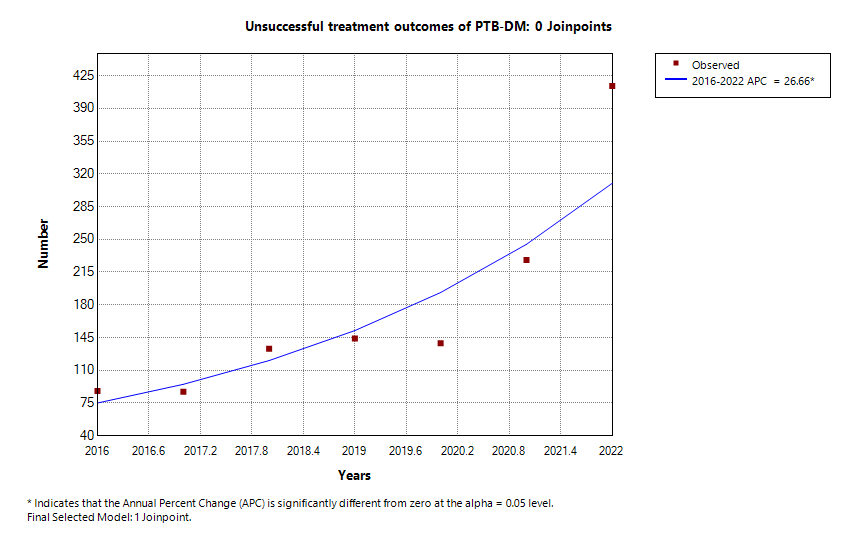 |
| --- | --- |
| 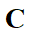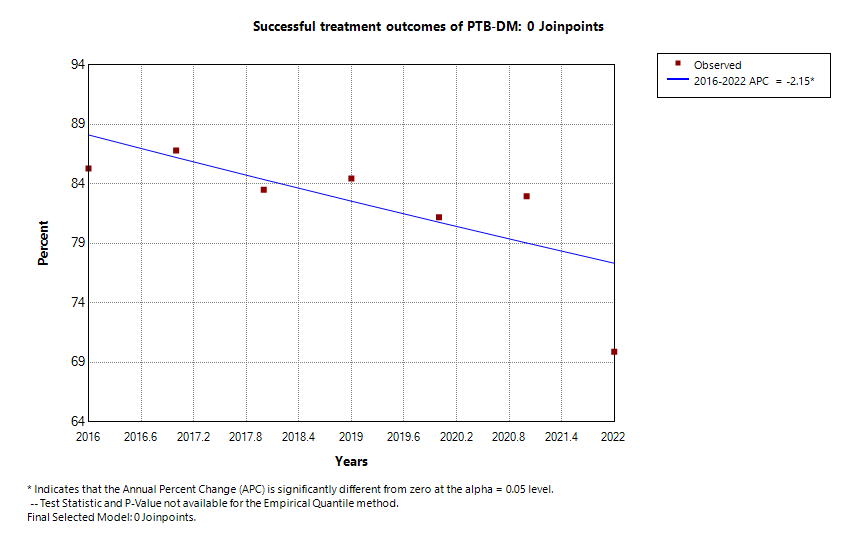 | 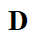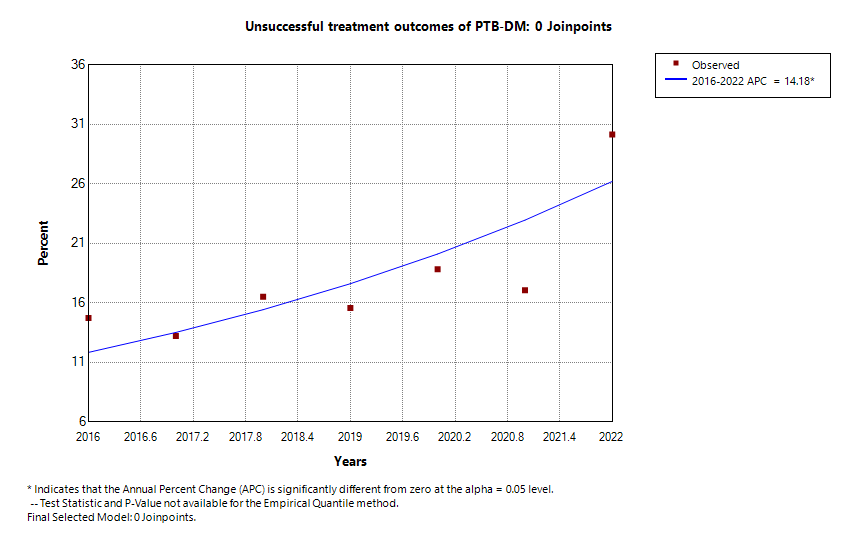 |

**Supplementary Figure 2.**Number and trends of successful and unsuccessful treatment outcomes of PTB-DM patients registered for treatment in Chongqing ,southwest China, 2016–2022.(A) Number of successful treatment outcomes. (B)Number of unsuccessful treatment outcomes. (C)Percentage of successful treatment outcomes. (D)Percentage of unsuccessful treatment outcomes .*Indicate that the Annual Percent Change (APC) is significantly different from zero at the alpha = 0.05 level .
